# Native Joint Septic Arthritis

**DOI:** 10.3390/antibiotics13070596

**Published:** 2024-06-27

**Authors:** Kevin A. Wu, David N. Kugelman, Jessica L. Seidelman, Thorsten M. Seyler

**Affiliations:** 1 Department of Orthopaedic Surgery, Duke University School of Medicine, Durham, NC 27701, USA; kevin.a.wu@duke.edu (K.A.W.);; 2 Division of Infectious Diseases, Duke University School of Medicine, Durham, NC 27710, USA

**Keywords:** septic arthritis, joint infection, antibiotic therapy, arthroscopic debridement, open surgical debridement, anterior cruciate ligament (ACL) reconstruction, rheumatoid arthritis, diagnosis, microbiology

## Abstract

Native joint septic arthritis (NJSA) is a severe and rapidly progressing joint infection, predominantly bacterial but also potentially fungal or viral, characterized by synovial membrane inflammation and joint damage, necessitating urgent and multidisciplinary management to prevent permanent joint damage and systemic sepsis. Common in large joints like knees, hips, shoulders, and elbows, NJSA's incidence is elevated in individuals with conditions like rheumatoid arthritis, diabetes, immunosuppression, joint replacement history, or intravenous drug use. This review provides a comprehensive overview of NJSA, encompassing its diagnosis, treatment, antibiotic therapy duration, and surgical interventions, as well as the comparison between arthroscopic and open debridement approaches. Additionally, it explores the unique challenges of managing NJSA in patients who have undergone graft anterior cruciate ligament (ACL) reconstruction. The epidemiology, risk factors, pathogenesis, microbiology, clinical manifestations, diagnosis, differential diagnosis, antibiotic treatment, surgical intervention, prevention, and prophylaxis of NJSA are discussed, highlighting the need for prompt diagnosis, aggressive treatment, and ongoing research to enhance patient outcomes.

## 1. Introduction

Native joint septic arthritis (NJSA) is a serious and fast-progressing infection of the joints. It is mainly caused by bacteria invading the joint space, although fungi and viruses can also be involved [1]. The condition causes inflammation of the synovial membrane and joint damage, making it a medical emergency. If not treated quickly, NJSA can lead to permanent joint damage and systemic sepsis. It usually affects large joints such as the knees, hips, shoulders, and elbows, with an incidence of 4–10 cases per 100,000 person-years. The risk is higher in people with conditions like rheumatoid arthritis, diabetes, immunosuppression, a history of joint replacement, or a history of intravenous drug use [2,3,4]. Even after antibiotic use, there is a 7–15% mortality rate for in-hospital septic arthritis [5].

The burden of NJSA is compounded by the aging population and increasing prevalence of comorbid conditions that predispose individuals to joint infections [6]. Recent studies have found that the incidence of NJSA has increased by a rate of 2–10 cases per 100,000 people per year [7].

This review aims to guide clinicians and researchers in effectively managing NJSA and to improve patient outcomes. We provide a comprehensive overview of NJSA, covering the critical aspects of its diagnosis, treatment, antibiotic therapy duration, and surgical interventions, as well as the comparison between arthroscopic versus open debridement approaches. Additionally, we explore the unique considerations of managing NJSA in patients who have undergone graft anterior cruciate ligament (ACL) reconstruction.

## 2. Epidemiology and Risk Factors

Identifying and addressing NJSA’s epidemiology and risk factors is vital for targeting at-risk populations and implementing prevention strategies. NJSA can affect individuals of all ages, but certain groups are more susceptible. Key risk factors include older age, pre-existing joint diseases such as rheumatoid arthritis or osteoarthritis, diabetes, immunosuppression, recent joint surgery, intravenous drug use, and systemic infections [8,9]. The increase in NJSA cases is partly due to an aging population and a rise in comorbid conditions [6]. Several potential strategies are highlighted in Table 1.

## 3. Pathogenesis and Microbiology

The development of NJSA is complex, involving the introduction of bacteria into the joint through various means such as hematogenous dissemination, direct inoculation, or contiguous spread from nearby infections [14]. Hematogenous dissemination, the most common pathway, occurs when bacteria travel through the bloodstream and settle in the synovial membrane, which lacks blood vessels but provides a nutrient-rich environment conducive to bacterial growth [15,16].

*Staphylococcus aureus* is the predominant pathogen responsible for approximately half of NJSA cases (Table 2) [9]. Other commonly isolated pathogens include various Streptococcus species, Gram-negative bacilli, and *Neisseria gonorrhoeae*, while anaerobic bacteria are less frequently found. Certain populations, such as intravenous drug users or immunocompromised individuals, may harbor atypical pathogens like *Pseudomonas aeruginosa* and fungi. Understanding the microbial landscape of NJSA is critical for selecting effective empirical antibiotics and tailoring treatment based on culture and sensitivity results.

Septic arthritis may rarely develop from an extension of an infection from nearby tissue [18]. Osteomyelitis within the metaphysis may break through the cortex of the bone and cause pus to discharge into the space [19]. This can occur in joints where the metaphysis is inside the capsular reflection (ex: knee, hips, shoulders, and elbows) or when hardware is placed in trauma sites.

Geographical variation in the organisms reflects regional differences in pathogen prevalence and antimicrobial resistance patterns. Staphylococcus aureus is a common culprit worldwide, but its prevalence can vary [20]. In tropical climates, Gram-negative organisms like *Salmonella* and *Pseudomonas aeruginosa* are more commonly implicated [21]. Additionally, the emergence of resistant strains, such as MRSA and vancomycin-resistant *enterococci* (VRE), presents challenges in managing NJSA. A recent study found that from 2019 to 2022, the prevalence of carbapenem-resistant microorganisms increased from 2.62% to 4.56%, while that of MRSA and VRE increased from 1.84% to 2.81% and 0.58% to 2.21%, respectively [22].

## 4. Clinical Manifestations

The clinical presentation of NJSA varies depending on the patient’s age, their overall health, and the infecting organism [6,23]. Common symptoms include joint pain, swelling, and fever, but patients may also experience chills, fatigue, and general malaise. Diagnosing NJSA in elderly patients can be particularly challenging due to their often milder and more subtle symptoms [24].

During a physical examination, typical signs of joint inflammation such as warmth, redness, and tenderness are usually observed [6,23,25]. The pain and swelling often limit joint movement and, in severe cases, can lead to deformity and impaired function. Early diagnosis and treatment are essential to prevent further joint damage and the spread of infection.

In children, NJSA may present differently, with signs such as irritability, refusal to use the affected limb, or limping [26,27]. Although fever and high inflammatory markers are common in pediatric cases, the signs of joint inflammation might be less apparent. Prompt identification and management are crucial to avoid long-term joint damage and chronic disability.

## 5. Diagnosis

Diagnosing NJSA requires careful attention due to its nonspecific symptoms [23]. A systematic review of 6242 patients found that the most common symptoms reported in patients with septic arthritis were joint pain, a history of joint swelling, and fever [28]. NJSA often presents suddenly with severe pain, swelling, limited joint movement, and tenderness [25]. Systemic symptoms such as chills, fever, and general discomfort may also occur, and sweats and rigors are less common. Because these symptoms can be similar to those of other joint issues, a thorough diagnostic workup is needed.

The main diagnostic procedure involves arthrocentesis to obtain synovial fluid, which is then examined with Gram staining and cultured to identify the causative organism. [16,29,30] Using blood culture bottles instead of agar plates for synovial fluid cultures offers several advantages, including broader pathogen identification, higher sensitivity, faster results, and lower sample quantity requirements [31,32,33]. However, this method is more expensive and may lead to more false-positive results, necessitating careful consideration [33].

Synovial fluid analysis is important to support the diagnosis of septic arthritis. Higher white blood cell (WBC) counts in the synovial fluid increase the likelihood of septic arthritis, and a high percentage of polymorphonuclear cells (90% or more) strongly indicates the condition [28]. Typically, synovial fluid in septic arthritis shows a WBC count over 50,000 cells per microliter [34]. Crystal analysis is also conducted to rule out conditions like gout or pseudogout. Other synovial markers, such as glucose and protein levels, are less reliable due to inconsistent sensitivity and specificity [28]. The leucocyte esterase (LE) strip testing of synovial fluid has also been explored as an option for diagnosing septic arthritis due to its speed, accuracy, and cost-effectiveness [35]. It can help differentiate septic arthritis from conditions like transient synovitis and juvenile rheumatoid arthritis and can be carried out at the bedside. Studies have demonstrated high specificity and sensitivity [35]. These tests also have a high negative predictive value, reducing time spent in the emergency department and hospital admissions. Additionally, synovial fluid lactate has demonstrated promise in differentiating between septic and non-septic arthritis, such as gouty arthritis and other inflammatory arthritides [36]. Elevated synovial lactate levels (>10 mmol/L) suggest septic arthritis, while lower levels (<4.3 mmol/L) are more indicative of gouty arthritis or other non-septic conditions [37]. However, caution is advised in interpreting results, especially in patients with recent antibiotic therapy or suspected gonococcal arthritis. Synovial fluid Alpha-defensin (αD), a neutrophil-released peptide, has been explored as a biomarker of infection [38]. However, its utility in diagnosing septic arthritis is questionable as some studies have demonstrated high rates of false positives and negatives [38,39].

Blood cultures are positive in about half of NJSA cases and help identify bacteremia [30]. Peripheral blood tests, including WBC count, erythrocyte sedimentation rate (ESR), and C-reactive protein (CRP), can support the diagnosis but are not specific [28,40,41,42]. Prior research has also examined the use of serum procalcitonin levels to differentiate between septic arthritis and gouty arthritis in patients with a history of gout [43]. Elevated procalcitonin levels (>0.81 ng/mL) showed 87% sensitivity and 95% specificity in differentiating septic arthritis from gout, suggesting its diagnostic utility in gout patients.

Imaging studies can also help diagnose NJSA [29]. X-rays may show joint effusion and soft tissue swelling, though they are not specific to NJSA and should not be used to diagnose NJSA since they are unable to provide additional information. Ultrasound is useful for detecting joint effusion and guiding arthrocentesis [44]. Although ultrasound is less expensive compared to modalities such as magnetic resonance imaging (MRI), it is unable to accurately assess the articular surface, which may limit its utility as a first-line imaging modality [45]. MRI, though more expensive, provides detailed images of joint structures and is useful for identifying early joint damage and soft tissue involvement [46]. While MRI images alone have been shown to present challenges in distinguishing septic arthritis, certain studies have indicated that patients with septic arthritis more frequently exhibit lamellated synovial thickening patterns, bone marrow edema, and soft tissue abscess formation than those with gouty arthritis [47]. In a meta-analysis, the diagnostic performance of MRI was assessed [45]. Synovial enhancement showed a pooled sensitivity of 94.2% (95% CI, 45.2–99.7%) and specificity of 60.6% (95% CI, 6–97.4%). Soft tissue changes demonstrated a sensitivity of 75% (95% CI, 57.5–86.9%) and specificity of 69.9% (95% CI, 46.5–86.2%) [45]. Femoral head changes had a sensitivity of 41.5% (95% CI, 15.9–72.7%) and specificity of 87.3% (95% CI, 75.5–93.8%) [45]. Lastly, bone marrow changes showed a sensitivity of 70% (95% CI, 26.8–93.7%) and an impressive specificity of 99.9% (95% CI, 28.7–100%) [45].

Improved diagnostic techniques have also led to better detection rates. Understanding these epidemiological trends helps clinicians identify high-risk groups and develop targeted interventions [48]. For instance, individuals with rheumatoid arthritis are at higher risk due to the disease and immunosuppressive treatments, highlighting the need for vigilant monitoring and early intervention.

Diagnosing NJSA in patients with underlying inflammatory arthritis such as rheumatoid arthritis or systemic lupus erythematosus presents significant challenges as these patients may be on biologic agents, corticosteroids, or other immunosuppressive therapies [11,49]. These treatments can mask typical signs of infection and alter inflammatory markers, complicating the clinical picture. During flare-ups of the underlying disease, distinguishing between an exacerbation of inflammatory arthritis and an acute septic arthritis can be difficult, as both conditions may present with increased joint pain, swelling, and systemic symptoms such as fever and elevated acute phase reactants [49]. Biologic agents and corticosteroids, while effective in controlling inflammation, suppress the immune system, increasing the risk of atypical and opportunistic infections, including those caused by less common pathogens such as the *Salmonella* species [50]. This further complicates the diagnostic process, as these infections may not present with classic symptoms and might be resistant to standard therapies. In such cases, a high index of suspicion is necessary, and joint aspiration with synovial fluid analysis remains the gold standard for diagnosis. Therefore, in patients with inflammatory arthritis on immunosuppressive therapy presenting with acute monoarthritis or polyarthritis, septic arthritis should be considered, with aggressive diagnostic workups conducted to differentiate it from an inflammatory flare. Collaboration among rheumatologists, infectious disease specialists, and orthopedic surgeons is often required to manage these complex cases effectively.

## 6. Differential Diagnosis

The differential diagnosis of NJSA includes various conditions with similar symptoms, such as crystal-induced arthritis (gout and pseudogout), reactive arthritis, rheumatoid arthritis, and trauma [51,52]. Distinguishing NJSA from these conditions is crucial due to the differing management approaches.

Crystal-induced arthritis can be excluded by analyzing synovial fluid crystals, showing monosodium urate crystals in gout and calcium pyrophosphate crystals in pseudogout [53]. The coexistence of crystalline arthropathy and septic arthritis complicates diagnosis, requiring empirical treatment for septic arthritis to avoid joint destruction. Reactive arthritis, linked to prior infections, manifests with arthritis, urethritis, and conjunctivitis [54]. Rheumatoid arthritis typically involves symmetrical joint damage and a chronic course, often with specific autoantibodies such as rheumatoid factor and anti-cyclic citrullinated peptide [11]. Additionally, rheumatoid arthritis can involve small joints not typically seen in NJSA.

Trauma history and imaging differentiate trauma-related joint issues from NJSA. Septic bursitis, mimicking NJSA, is identified through clinical examination and imaging [52,55]. Other inflammatory arthritides like psoriatic arthritis and ankylosing spondylitis should also be considered based on clinical history and specific diagnostic criteria [56,57].

Hypersensitivity reactions to injected agents, such as hyaluronan, can cause symptoms similar to those of septic arthritis, including joint pain and swelling [58]. Additionally, bone conditions like Gorham disease, SAPHO syndrome, chronic recurrent multifocal osteomyelitis, Paget disease, systemic mastocytosis, and synovial chondromatosis can present with atypical and challenging clinical manifestations [30].

## 7. Antibiotic Treatment

The treatment approach for NJSA aims to eradicate the infection, alleviate pain, and preserve joint function [16]. It typically involves a combination of antibiotic therapy and, in many cases, surgical intervention. Antibiotic treatment is initiated promptly after obtaining synovial fluid and blood cultures, often starting with broad-spectrum antibiotics such as vancomycin [59]. For immunocompromised patients or those with a history of intravenous drug use, a third-generation cephalosporin may be added. Treatment is adjusted based on culture results and sensitivity testing, with *Staphylococcus aureus*, particularly methicillin-resistant *Staphylococcus aureus* (MRSA), commonly implicated.

The optimal duration of antibiotic therapy for septic arthritis, including NJSA, remains uncertain and is often individualized based on clinical judgment and patient factors (Table 3). However, guidelines generally recommend a 4–6-week course of antibiotics, tailored to factors such as infection severity, causative organism, and patient response [59]. Fungal infections may require longer durations. Concurrent bacteremia, endocarditis, and osteomyelitis can also prolong therapy. The initial phase often involves 2–3 weeks of intravenous antibiotics to achieve high serum and synovial fluid antibiotic concentrations for rapid infection control, followed by oral antibiotics to complete the course. Some studies suggest that shorter courses of antibiotics may be effective [60,61].

For MRSA or methicillin-susceptible *Staphylococcus aureus* (MSSA), treatment typically involves at least 14 days of parenteral therapy followed by 1–2 weeks of oral therapy. *Gonorrhea* is commonly treated with ceftriaxone for 14 days. The duration for other bacteria varies, but 3–4 weeks is generally sufficient, with a switch to oral therapy if suitable and patient improvement is observed. Regular follow-up visits are essential to monitor clinical response, therapy side effects, and laboratory markers, with adjustments to the antibiotic regimen based on culture results and patient progress [62].

Adjunctive therapies are crucial in managing NJSA. Pain is typically managed with nonsteroidal anti-inflammatory drugs (NSAIDs) or acetaminophen [6,63]. Early mobilization and physical therapy are essential to maintain joint function and prevent stiffness after controlling the acute infection [64].

Complex cases can also involve unusual types of bacteria, such as *Ureaplasma urealyticum*, which can acquire resistance in patients treated with biologic agents that suppress the B-cell population [65,66]. This adds another layer of complexity to the diagnosis and treatment, as these atypical infections may not respond to standard antibiotic regimens and require specialized management.

## 8. Surgical Intervention

Surgical intervention is often necessary in NJSA, especially in cases of severe infection, failure of medical management, or significant joint destruction [67,68]. Criteria for surgery include persistent or worsening symptoms despite antibiotics, presence of joint effusion or abscess, joint instability, compromised joint function, and systemic symptoms [67,68]. The primary goals of surgery are to remove infected material, reduce the bacterial load, and preserve joint function. Arthroscopic debridement is a less invasive procedure that allows the direct visualization and lavage of the joint [69,70,71,72]. It is preferred in early and less severe cases, involving multiple small incisions through which a camera and instruments are inserted to wash out the joint and remove infected tissue. The advantages of arthroscopic debridement include shorter recovery time, reduced postoperative pain, and a lower risk of complications compared to open surgery.

Open surgical debridement involves a larger incision, providing better access to the joint for thorough debridement and drainage [73]. It is indicated for severe infections, the presence of joint abscesses, or when arthroscopic debridement fails. The joint is opened, infected tissue is removed, and the joint is thoroughly irrigated. Open surgical debridement offers a more comprehensive removal of infected material and is suitable for complex cases, though it is more invasive, leading to a longer recovery time and a higher risk of postoperative complications.

In cases of irreparable joint damage or in patients with pre-existing joint prostheses, joint arthroplasty may be necessary [74,75,76]. This can involve partial or total joint replacement and is considered when conservative measures and debridement fail to preserve joint function or when there is extensive joint destruction.

Postoperative care aims to prevent complications, promote healing, and restore joint function. This includes continued or adjusted antibiotic therapy, joint immobilization, early physical therapy, monitoring for complications, and patient education. Regular monitoring postoperatively is crucial to detect and manage complications early.

## 9. Arthroscopic versus Open Surgical Debridement

The decision between arthroscopic and open surgical debridement depends on several factors, including the extent of infection, the patient’s overall health, and the surgeon’s expertise [70]. Both approaches have their advantages and disadvantages. Arthroscopic debridement is minimally invasive, resulting in less soft tissue damage, a shorter recovery time, and reduced postoperative pain [77,78]. It also carries a lower risk of complications such as infection and thromboembolism. However, it may provide limited access in cases of extensive infection or anatomical abnormalities and may not be sufficient for severe or chronic infections.

Open surgical debridement provides better access for thorough debridement and drainage, making it suitable for complex cases with extensive infection. However, it is more invasive, leading to a longer recovery time and a higher risk of postoperative complications, including infection and thromboembolism.

Dobek et al. found in a national insurance database study that there was no significant difference in reoperation rate between arthroscopic and open debridement (15.0.% open and 18.0% arthroscopic, *p* = 0.174) [79]. However, they found that those who had open debridement had an increased readmission (OR = 1.46 [1.14–1.86]; *p* = 0.003) and blood transfusion (OR = 1.76 [1.04–3.06]; *p* = 0.040) rate compared to those who underwent arthroscopic debridement [79]. A review found that these two surgical techniques had similar outcomes [70]. They found that reoperation rates varied widely, from 0% to 50% for arthroscopy cases and 6% to 71% for arthrotomy cases [70]. Complication rates also varied, from 0% to 39.4% for arthroscopy cases and 0% to 49% for arthrotomy cases. Two studies included in the review reported superior subjective patient-reported outcomes with arthroscopy [70].

## 10. Native Joint Septic Arthritis in the Context of Graft ACL Reconstruction

Managing NJSA in patients with graft ACL reconstruction presents unique challenges. The presence of the graft introduces a foreign material into the joint, which can serve as a potential nidus for infection and complicate the treatment course. The immune response to the graft may also be altered, potentially affecting the presentation and progression of the infection. Furthermore, the rehabilitation process following ACLR can mask or delay the recognition of symptoms of NJSA, leading to a delay in diagnosis and treatment initiation. Infections can lead to graft failure and compromise the overall outcome of the surgery [80,81,82]. Diagnosing NJSA in these patients requires a higher index of suspicion due to altered anatomy and the potential masking of symptoms through postoperative changes [83]. MRI is particularly useful for detecting infection around the graft site and assessing the extent of involvement.

The treatment of NJSA in the context of graft ACL reconstruction involves the prompt initiation of targeted antibiotic therapy to control the infection and prevent graft failure. Decisions on whether to preserve or remove the graft depend on the severity of the infection and graft stability [81]. MRI can be useful for detecting infection around the graft site and assessing the extent of involvement [84]. Additionally, MRI can help differentiate between infectious and non-infectious causes of graft failure, aiding in treatment decisions. In some cases, the debridement and retention of the graft are feasible, while in others, graft removal and subsequent staged reconstruction may be necessary. Graft removal has demonstrated superior results to graft retention, and in cases in which NJSA is high in the differential, graft removal should be pursued [85].

Surgical management often involves arthroscopic debridement to minimize additional surgical trauma and facilitate early recovery. A study of 31 military personal conducted by Waterman et al. found that 48% of patients returned to military duty following arthroscopic debridement at an average of 26.9 months of follow-up. However, in severe cases or when arthroscopic debridement is insufficient, open surgical debridement may be necessary.

## 11. Prevention and Prophylaxis

Preventing NJSA involves addressing modifiable risk factors and implementing prophylactic measures in high-risk populations [59]. Patients with joint prostheses or those undergoing joint surgery should receive perioperative antibiotic prophylaxis to reduce the risk of infection. Preoperative antibiotics are justified in cases where the patient has a history of septic arthritis or an inflammatory disorder. However, it is important to be mindful of preoperative antibiotic use as it increases the risk of antibiotic resistance. Following strict aseptic techniques during joint injections and surgeries is crucial in preventing iatrogenic infections [86].

For individuals with recurrent NJSA or underlying conditions predisposing them to joint infections, long-term antibiotic prophylaxis may be considered. Vaccination against pathogens such as *Streptococcus pneumoniae* and influenza can reduce the risk of hematogenous spread of these organisms to the joints [87].

## 12. Discussion

NJSA demands prompt recognition, along with aggressive treatment, to prevent joint destruction and systemic complications. Its complexity lies in its potential to rapidly cause irreversible damage, necessitating an urgent, multidisciplinary management approach [6,23]. Advances in diagnostic techniques and therapeutic strategies in recent years have substantially improved patient outcomes [23].

Preventing native joint septic arthritis involves several key strategies [5,10,11,13]. The prompt treatment of skin and respiratory infections, especially in patients with chronic conditions like diabetes or rheumatoid arthritis, can prevent the spread of bacteria to the joints. Ensuring strict aseptic techniques during medical procedures and the proper management of intravenous catheters minimize the risk of introducing bacteria into the joint space. Strict glucose control in diabetic patients and the careful management of rheumatoid arthritis with disease-modifying drugs can reduce the overall risk of infections. Vaccination against common pathogens and promoting good personal hygiene practices also play crucial roles in prevention. Educating patients on the symptoms of septic arthritis and the importance of early medical consultation can lead to quicker diagnosis and treatment, preventing severe joint damage. Lifestyle modifications, such as maintaining a healthy diet and avoiding high-risk behaviors, strengthen the immune system and reduce the risk of infections. By implementing these strategies, the incidence of native joint septic arthritis can be reduced, leading to better health outcomes and the preservation of joint function.

Imaging technologies like MRI and ultrasound have improved the early identification of joint effusions and synovial inflammation and aided in guiding diagnostic arthrocentesis [46,88]. The two modalities differ in cost, and the decision to pursue one or the other should depend on the suspicion of the treating provider. Molecular diagnostic tools, including PCR-based assays, offer the rapid and accurate identification of causative pathogens, especially in cases where traditional cultures are inconclusive. Diagnosing NSJA in patients on immune modulating medications remains a challenge, as the medications may mask some of the common symptoms which may alert providers.

Treatment strategies have evolved, offering a better understanding of the need for both medical and surgical interventions. Antibiotic therapy remains crucial, with a more sophisticated approach considering local resistance patterns and patient-specific factors [89]. The duration of antibiotic therapy is tailored to the patient’s response, aiming for the complete eradication of the infection while minimizing the risk of resistance and adverse effects.

Surgical interventions, including arthroscopic and open surgical debridement, play a crucial role in managing NJSA, particularly in severe or refractory cases. Arthroscopic surgery, being minimally invasive, allows for effective drainage and debridement with reduced recovery times and low complication rates compared to traditional open surgery [68]. However, open surgical intervention remains indispensable in cases of extensive joint destruction or when arthroscopic techniques are insufficient.

Managing NJSA in the context of graft ACL reconstruction presents unique challenges as the presence of a graft can serve as a nidus for persistent infection [80,81,82,83]. Preserving the graft’s integrity to ensure successful long-term outcomes requires special considerations and may involve more aggressive surgical debridement with a prolonged course of tailored antibiotic therapy. In cases where there is a high suspicion of NJSA, the graft should be removed to preserve the cartilage, as studies have demonstrated superior outcomes through this method. It is important to achieve a balance between eradicating the infection and preserving graft function [81].

Despite these advancements, there are still significant gaps in our understanding and management of NJSA. Further research is needed to optimize treatment protocols and improve prognostic indicators. Identifying biomarkers predicting disease severity and treatment response could guide more personalized therapeutic approaches [90].

Future research in NJSA should focus on several key areas to continue improving outcomes for patients. This includes developing and validating novel diagnostic tools allowing for the earlier and more accurate detection of joint infections. Potential areas include techniques such as next-generation sequencing to identify pathogens even in culture-negative cases [91,92]. Developing new therapeutic agents and antibiotics is also critical to effectively target resistant organisms [93]. Additionally, the development of adjunctive therapies that modulate the host’s immune response could enhance treatment outcomes. For example, anti-inflammatory agents that specifically target the inflammatory pathways involved in joint destruction may help preserve joint function while the infection is being treated. Understanding the pathogenesis of NJSA at a molecular level is essential for developing targeted therapies [94,95]. Research into how bacteria evade the host immune response and persist in the joint environment can inform new strategies to prevent joint destruction and recurrence of infection. This deeper understanding of host–pathogen interaction could lead to the discovery of novel therapeutic targets [96,97]. Preventive strategies are another important area of research. Vaccines against common causative organisms of NJSA could significantly reduce the incidence of this condition [87]. Research into immunomodulatory therapies that enhance the host’s ability to fight off infections without causing excessive inflammation is also essential.

Additionally, future research should focus on developing standardized treatment algorithms and determining the optimal duration of antibiotic therapy. Currently, there is a lack of consensus regarding the most effective treatment approach for NJSA, leading to variability in clinical practice. Establishing evidence-based algorithms that consider factors such as the causative organism, the severity of the infection, and patient-specific factors could help standardize treatment and improve outcomes. Additionally, determining the optimal duration of antibiotic therapy is crucial to balance the need for adequate infection control with the risk of antibiotic-related complications, such as resistance and adverse effects. Future research should aim to conduct well-designed clinical trials to address these gaps in knowledge and provide clear guidance for clinicians managing NJSA.

Investigating the long-term outcomes of patients with NJSA and identifying factors associated with poor prognosis can help develop personalized treatment plans. This includes understanding the impact of NJSA on joint function, quality of life, and the risk of recurrent infections. Longitudinal studies that follow patients over time can provide valuable insights into the natural history of NJSA and the effectiveness of different treatment strategies.

Increasing awareness among healthcare providers and patients about the signs and symptoms of NJSA can lead to earlier diagnosis and treatment. Educational initiatives that emphasize the importance of seeking medical attention for joint pain and swelling, particularly in high-risk populations, can improve outcomes [98].

Developing effective treatment options for NJSA also has implications for the management of periprosthetic joint infection (PJI) following arthroplasty [99]. PJI is a significant complication of arthroplasty, leading to increased healthcare costs and patient morbidity [12,99,100,101,102]. By improving our understanding and treatment strategies for NJSA, we can potentially reduce the incidence and severity of PJI, thus improving patient outcomes and reducing the economic burden associated with these infections.

The management of NJSA often requires a multidisciplinary approach involving rheumatologists, infectious disease specialists, orthopedic surgeons, and physical therapists [103]. Collaborative care models that integrate these specialties can optimize patient outcomes by providing comprehensive and coordinated care. NJSA remains a challenging condition to manage, but ongoing research and advancements in diagnostic and therapeutic modalities hold promise for improving patient outcomes. By addressing the current gaps in knowledge and focusing on personalized, evidence-based approaches, we can continue to enhance the care and prognosis for patients with NJSA. The integration of novel diagnostic tools, therapeutic agents, and preventive strategies into clinical practice will be key in reducing the burden of this serious condition and improving the quality of life for affected individuals.

## Figures and Tables

**Table 1:** Prevention strategies for native joint septic arthritis.

Prevention Strategy	Description
Prompt treatment of infections	Immediate and effective treatment of skin and respiratory infections to prevent spread to joints.
Aseptic techniques in medical procedures	Ensuring strict aseptic techniques during joint injections, surgeries, and catheter use [10].
Management of chronic conditions	Strict glucose control in diabetes and careful management of rheumatoid arthritis [11,12].
Immunization	Vaccination against pathogens like *Streptococcus pneumoniae* and *Haemophilus influenzae* [13].
Hygiene and wound care	Encouraging good personal hygiene and proper care and treatment of wounds and cuts [5].
Awareness and early diagnosis	Educating patients on symptoms and training healthcare providers to recognize early signs.
Lifestyle modifications	Promoting a healthy lifestyle and advising against high-risk behaviors that lead to joint injuries [5].

**Table 2:** Common organisms and populations.

Organism	Population
*Staph aureus*	Healthy patients, arthroplasty patients, those with a history of cartilage damage
*Staph epidermidis*	Arthroplasty patients and post-surgical patients (ex: arthroscopy)
*Streptococci*	Patients with a nonfunctional spleen (ex: sickle cell, splenic removal)
*Neisseria gonorrhea*	Sexually active patients
Gram-negative rods: *E. Coli*, *Pseudomonas, Serratia*	Intravenous drug users, patients with immunosuppression (ex: chemotherapy, HIV), elderly patients
Information adapted from [17].	

**Table 3:** Antibiotic interval based on organism.

Organism	Antibiotic Interval
MRSA/MSSA	14 days of parenteral Additional 7 to 14 days of oral (if amenable)
Gonorrhea	Ceftriaxone Duration is at least 14 days
Other Bacteria	3–4 weeks total Can switch to oral after 5–7 days of parenteral therapy if able to use fluoroquinolone or other highly bioavailable agent and patient clinically improving

Abbreviations: MSSA: methicillin-susceptible *Staphylococcus aureus*; MRSA: methicillin-resistant *Staphylococcus aureus*.

## Data Availability

No new data were created or analyzed in this study. Data sharing is not applicable to this article.
